# Necrotizing Fasciitis Caused by Pseudomonas aeruginosa in an Immunocompromised Patient: A Case With Fulminant Evolution

**DOI:** 10.7759/cureus.91349

**Published:** 2025-08-31

**Authors:** Vera Vieira, Leonor Simões, Ana Nogueira, Ana Marques, Andreia Santos

**Affiliations:** 1 Intensive Care Unit, Unidade Local de Saúde da Região de Leiria, Leiria, PRT; 2 Intensive Care Unit, Unidade Local de Saúde de Coimbra, Coimbra, PRT

**Keywords:** critical care, immunosuppression, necrotizing fasciitis, pseudomonas aeruginosa, septic shock, soft tissue infection

## Abstract

Necrotizing fasciitis is a rare, life-threatening infection involving the subcutaneous tissue and fascia, with potential for rapid progression and high mortality. Here, we report the case of a 56-year-old woman with a history of chronic lymphocytic leukemia (under watchful waiting), hypogammaglobulinemia, and megaloblastic anemia who was admitted to the emergency department with progressive swelling of her right forearm following an insect bite. The patient rapidly developed septic shock with multiorgan failure. Broad-spectrum antibiotics were started, and she underwent urgent surgical debridement. Despite intensive care measures, the patient died within hours of admission. Cultures from the wound and blood grew *Pseudomonas aeruginosa*. This case illustrates the extreme virulence of *Pseudomonas aeruginosa* in necrotizing soft tissue infections, particularly in immunocompromised hosts. Early recognition and aggressive multidisciplinary intervention remain key to improving outcomes in this life-threatening condition.

## Introduction

Necrotizing fasciitis (NF) is an infection of the subcutaneous tissue and fascia. Even though it is rare, it can be life-threatening with fulminant progression and high mortality [1-4]. It affects mostly the limbs (upper and lower), abdominal wall, and perineum [1,3].

The most common agents implicated are group A *Streptococcus* and polymicrobial infections [1]. *Pseudomonas aeruginosa* is an exceptionally rare but increasingly isolated causative agent, especially in immunocompromised patients and in hospital-acquired infections [2,3,5,6]. The incidence of monomicrobial NF has been increasing [2]. Only a few cases of NF caused by *Pseudomonas aeruginosa* have been reported in the literature [2,7]. Common predisposing factors include diabetes, malignancy, alcohol misuse, and chronic liver and renal disease [1,5-7].

The diagnosis is based on clinical criteria [4]. Initial symptoms are usually pain, erythema, swelling, and fever [1]. Discomfort that first manifests out of proportion to the clinical findings is a defining characteristic [1].

NF is classified into the following four types based on the organism isolated on the Gram stain and culture: type 1 (80-90% of the infections) is polymicrobial, caused by non-Group A *Streptococcus* with aerobes and anaerobes; type 2 is caused by Group A-beta hemolytic streptococci; type 3 is caused by marine Gram-negative rods (e.g., *Vibrio* bacteria and *Pseudomonas aeruginosa*); and type 4, which is extremely rare, is caused by fungal infections (mainly *Aspergillus*, *Zygomycetes*, *Candida*, *Mucor*, and *Rhizopus* species) [4,5].

The speed of progression of the infection is directly proportional to the thickness of the subcutaneous tissue [5]. Rapid diagnosis, surgical debridement, and intensive care support are crucial for the prognosis [5-7]. The major indicator for the diagnosis of NF is the finding of necrosis of the fascial planes during surgery [4]. Aggressive surgical debridement is the most effective method for reducing mortality rates [4].

Blood purification therapies may help restore immune function by clearing inflammatory mediators from plasma, as well as improving oxygenation and hemodynamics [7], but the evidence is weak [8]. Plasmapheresis could be considered as an adjunct therapy in cases caused by pathogens known to produce toxins (clostridial, streptococcal, or staphylococcal infections) and never as a replacement for aggressive surgical debridement. Continuous renal replacement therapy is the preferred mode in sepsis-induced acute kidney injury [7].

This report describes a fulminant case of NF caused by *Pseudomonas aeruginosa* in a patient with chronic lymphocytic leukemia and hypogammaglobulinemia, highlighting the importance of considering atypical pathogens in immunocompromised patients.

## Case presentation

**Figure 1:**
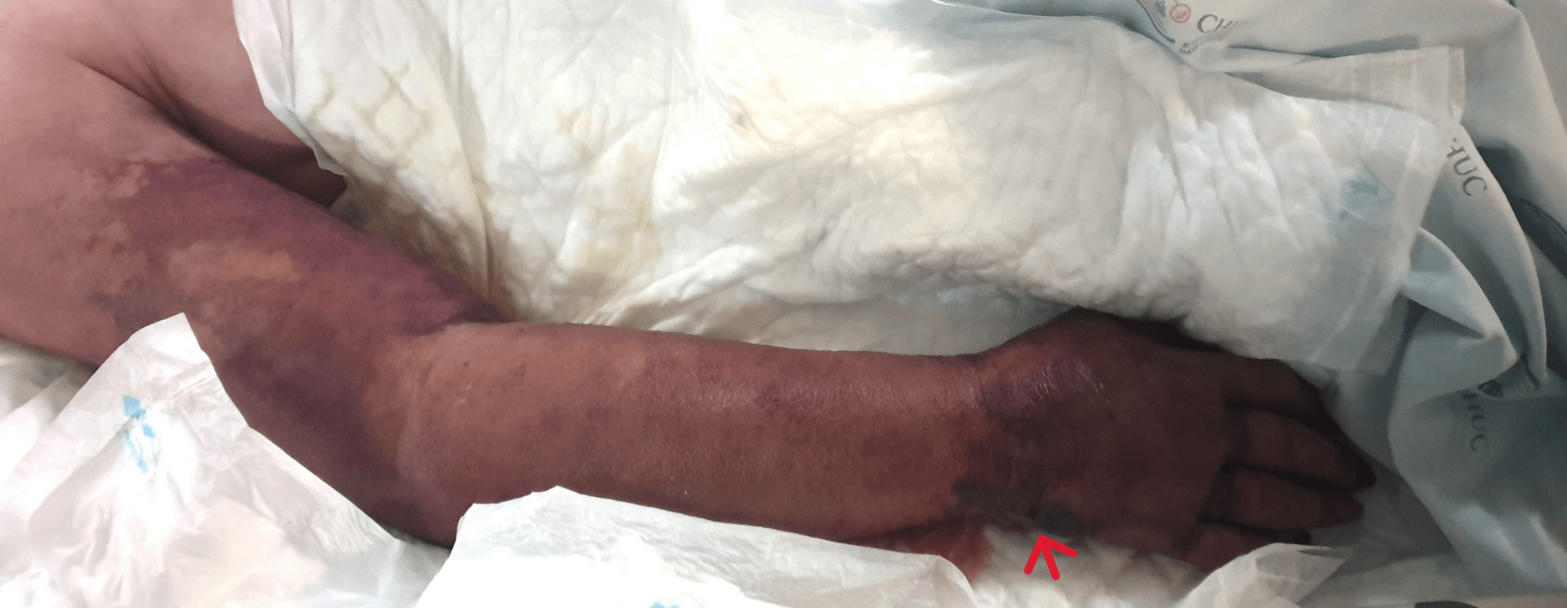
Patient’s right upper limb upon admission to the Intensive Care Unit before the surgical exploration and debridement showing edema, violaceous erythema, and some hemorrhagic bullae on the back of hand and wrist.

A 56-year-old female, autonomous in daily activities, with chronic lymphocytic leukemia under surveillance, hypogammaglobulinemia, and megaloblastic anemia, presented to the emergency department with rapid-onset swelling and erythema of the right forearm (Figure 1).

She reported an insect bite in the same region the day before. The patient had been discharged two days earlier from a hospital stay for infectious diarrhea (treated with azithromycin for eight days), with stool cultures positive for *Campylobacter*, *Astrovirus*, and *Norovirus* GI/GII.

**Figure 2:**
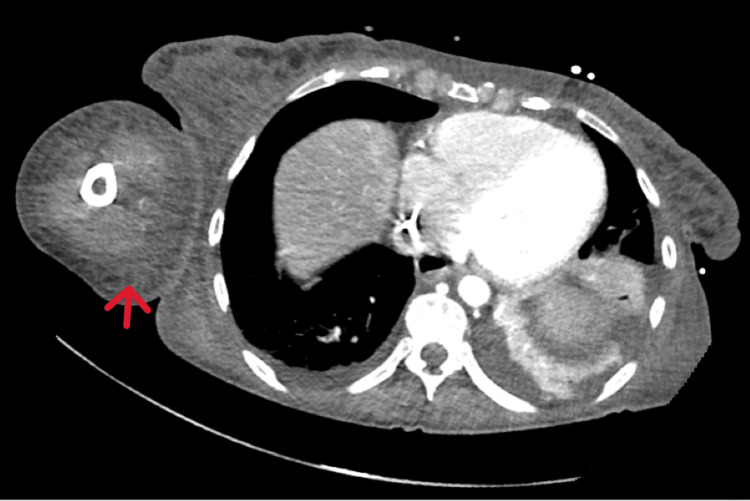
Contrast-enhanced CT scan showing thickening of the skin and subcutaneous tissue of the right arm and forearm of nonspecific appearance.

Venous Doppler excluded deep vein thrombosis. Contrast-enhanced CT scan revealed patency of the right subclavian vein but noted stenosis of the right brachiocephalic vein and partial thrombosis of the distal superior vena cava (25 mm), along with diffuse skin and subcutaneous thickening in the right arm and forearm (Figure 2).

Despite initial stability, the patient rapidly progressed to being prostrated, hypotensive, and unresponsive to crystalloid infusion. An echocardiogram showed signs of compromised left ventricular function, which was admitted in the septic context. Clinical evolution and laboratory findings supported the diagnosis of septic shock with multiorgan dysfunction (cardiovascular, neurologic, renal, hepatic) (Table 1).

**Table 1:** Patient’s labs on presentation.

Parameter	Result
White blood cells, /mL	3,900
Hemoglobin, g/dL	10.1
Platelets, 10^9^/L	66,000
Prothrombinemia, %	38
Sodium, mmol/L	127
Aspartate aminotransferase, U/L	107
Alanine aminotransferase, U/L	57
Creatinine, mg/dL	1.53
C-reactive protein, mg/L	250
Glucose, mg/dL	99
N-terminal pro-B-type natriuretic peptide, pg/mL	21,082
Blood gas (FiO_2_ 50%)	
pH	7.5
pCO_2_, mmHg	20.3
pO_2_, mmHg	98.2
Bicarbonate, mEq/L	15.5
Lactate, mmol/L	6.23

Empiric antibiotics (vancomycin, meropenem, and clindamycin) were initiated. She underwent urgent surgical debridement of the right upper limb, which showed necrosis of the fascial planes. Vasopressor support was escalated, intravenous hydrocortisone was administered, and continuous venovenous hemodiafiltration with cytokine hemoadsorption was initiated.

Despite early antibiotics, surgical debridement, and maximal supportive therapy, the patient died within hours of Intensive Care Unit admission. Blood cultures and wound aspirates were positive for *Pseudomonas aeruginosa*.

## Discussion

This case demonstrates the potential for *Pseudomonas aeruginosa* to cause aggressive necrotizing soft tissue infections, especially in patients with compromised immunity. The patient’s underlying hematologic malignancy and hypogammaglobulinemia likely contributed to both the rapid progression and poor outcome.

*Pseudomonas aeruginosa* is more commonly associated with nosocomial infections. Its role in NF, though rare, has been increasingly reported, particularly in immunocompromised individuals. The bacteria’s virulence factors (pili, lipopolysaccharide, exotoxins, proteases, *Pseudomonas aeruginosa* elastase, and biofilm formation) contribute to the rapid tissue spread and resistance to the host’s defense mechanisms [2,3]. The virulence factors of *Pseudomonas aeruginosa* compromise leucocyte adherence, chemotaxis, intracellular bacterial killing, antigen-specific cell-mediated immunity, and proliferative responses, leading to severe skin and soft tissue damage, which enables *Pseudomonas aeruginos*a to infect deeper and cause bacteremia [2,3].

Early diagnosis is often extremely challenging due to nonspecific initial presentations. Imaging findings may be subtle; therefore, clinical suspicion is crucial. Clinicians should always think of atypical pathogens, especially in immunocompromised patients. Once NF is suspected, prompt surgical exploration and debridement, as well as broad-spectrum antibiotics and intensive care support, are essential. Even with optimal management, outcomes may be poor in fulminant cases. Hyperbaric oxygen has been suggested by the literature as an adjunct therapy, but our patient had a fulminant evolution, and that option was not possible.

## Conclusions

NF caused by *Pseudomonas aeruginosa* is rare but should be considered in immunocompromised patients presenting with rapidly progressive soft tissue infections. This case highlights the need for early clinical suspicion, prompt surgical intervention, and intensive care management to attempt to alter the typically fatal prognosis. The need for increased awareness, reporting, and research on atypical pathogens in NF is crucial.
